# The interplay of APOE and APOA1 gene polymorphisms modulates the risk of type 2 diabetes mellitus in an obese population: a case–control study"

**DOI:** 10.1186/s40001-025-03829-0

**Published:** 2026-01-27

**Authors:** Nagla Usama, Amr E. Ahmed, Salma Mekheimer, Khaled Elhadidy, Mahmoud Farid

**Affiliations:** 1 Medical Laboratory Technology Department, Faculty of Applied Health Science Technology, Misr University for Science and Technology, Cairo, Egypt; 2 Biotechnology and Life Sciences Department, Faculty of Postgraduate Studies for Advanced Sciences, Beni-Suef University, Beni-Suef, Egypt; 3 Internal Medicine Department at Faculty of Medicine, Beni-Suef University, Beni-Suef, Egypt

**Keywords:** Obesity, Type 2 diabetes mellitus, *APOE*, *APOA1*, Genotyping

## Abstract

**Background:**

Genetic factors play an important role in metabolic disease susceptibility. Apolipoproteins E (*APOE*) and A1 (*APOA1*) are key regulators of lipid metabolism and have been individually associated with dyslipidemia and type 2 diabetes mellitus (T2DM).

**Objective:**

This study aimed to examine the individual and combined associations of *APOE* (rs429358, rs7412) and *APOA1* (rs5069) gene polymorphisms with obesity and T2DM.

**Methods:**

A case–control study was conducted including 350 participants categorized into four groups: controls (n = 100), euglycemic obese individuals (n = 100), obese individuals with T2DM (n = 100), and non-obese individuals with T2DM (n = 50). Biochemical parameters, including lipid profiles and glycemic indices, were assessed. Genotyping was performed using TaqMan^®^ SNP genotyping assays.

**Results:**

Metabolic disturbances and dyslipidemia were observed across all patient groups, with the most pronounced abnormalities in obese individuals with T2DM. The *APOE* ε4 allele and ε4/ε4 genotype were significantly associated with obese T2DM compared with controls and euglycemic obese subjects. The *APOA1* rs5069 A allele and AA genotype were associated with both obesity and T2DM. Spearman correlation analysis revealed a positive co-occurrence of *APOE* and *APOA1* genotypes in euglycemic obese (ρ = 0.264, p = 0.008) and obese T2DM (ρ = 0.347, p < 0.001) groups, but not in non-obese T2DM individuals. However, in multivariate logistic regression models adjusted for age, sex, and BMI, the *APOE* × *APOA1* interaction term did not reach statistical significance (p = 0.138).

**Conclusion:**

*APOE* ε4 and *APOA1* rs5069 A alleles were independently associated with obesity-related T2DM. Although these variants demonstrated correlated distribution patterns in obese individuals, the formal gene–gene interaction on T2DM risk was not statistically significant after multivariable adjustment. These findings suggest that obesity may represent a metabolic context in which combined genetic associations are more evident, warranting further investigation in larger and well-powered cohorts.

**Supplementary Information:**

The online version contains supplementary material available at 10.1186/s40001-025-03829-0.

## Introduction

Obesity and type 2 diabetes mellitus (T2DM) represent major global public health challenges, with a steadily rising prevalence that contributes substantially to cardiovascular morbidity and mortality [1]. Both conditions are closely associated with insulin resistance, dyslipidemia, and chronic low-grade inflammation, which together constitute core components of the metabolic syndrome [2].

Apolipoproteins play a central role in lipid metabolism, a process frequently disrupted in metabolic disorders [3]. Apolipoprotein E (*APOE*), a key ligand involved in lipoprotein receptor–mediated clearance, exists in three major isoforms (ε2, ε3, and ε4) encoded by polymorphisms in the *APOE* gene (rs429358 and rs7412) [4]. Two coding single-nucleotide polymorphisms (SNPs) in exon 4 of *APOE*—rs429358 (T → C) and rs7412 (C → T)—determine these isoforms. Specifically, ε2 corresponds to rs429358-T/rs7412-T (Cys112, Cys158), ε3 to rs429358-T/rs7412-C (Cys112, Arg158), and ε4 to rs429358-C/rs7412-C (Arg112, Arg158). These structural differences influence receptor binding affinity and lipid transport behavior, contributing to interindividual variability in lipid metabolism and disease susceptibility [5]. The ε4 allele has been consistently associated with hypercholesterolemia and cardiovascular disease, and accumulating evidence suggests that *APOE* polymorphisms may also be associated with insulin sensitivity and T2DM risk, although findings across populations remain heterogeneous.

Apolipoprotein A1 (*APOA1*), the principal protein component of high-density lipoprotein (HDL), is essential for reverse cholesterol transport and endothelial function [6]. Genetic variation within the *APOA1* gene, including the rs5069 polymorphism, has been associated with differences in HDL cholesterol levels and functionality, suggesting potential links to insulin resistance and diabetes [7].

These metabolic pathways do not operate independently. The close relationship between dyslipidemia and insulin resistance is well established, with obesity acting as a shared metabolic context in which both abnormalities frequently coexist and intensify [8]. In this setting, genetic variation in lipid-related genes such as *APOE* and *APOA1* may show coordinated distribution patterns or context-dependent associations with metabolic traits [9, 10]. *APOE* and *APOA1* were selected for the present study because they occupy complementary roles in lipid metabolism and inflammation: *APOE* variants influence the clearance of triglyceride-rich lipoproteins and adipose tissue immunometabolism, whereas *APOA1* rs5069 has been linked to alterations in HDL structure and function. Obesity amplifies metabolic pathways governed by both genes, making their combined evaluation particularly relevant for obesity-related T2DM. Despite extensive literature examining each gene individually, their combined patterns of association within obesogenic metabolic environments especially in Middle Eastern and North African populations remain relatively underexplored, providing a rationale for the present investigation [11, 12].

Accordingly, this case–control study employed a multi-group design to address two main objectives: first, to examine whether polymorphisms in *APOE* and *APOA1* are independently associated with obesity and T2DM; and second, to explore whether patterns of co-occurrence or interaction between these variants differ according to obesity status. By comparing healthy controls with euglycemic obese, obese T2DM, and non-obese T2DM individuals, we aimed to assess the individual and combined associations of *APOE* (rs429358, rs7412) and *APOA1* (rs5069) polymorphisms with obesity and T2DM.

## Materials and methods

### Study design and population

A case–control study was conducted at the Diabetic Clinic of Memorial Souad Kafafi University Hospital, Egypt. A total of 350 participants aged 35–55 years were recruited and classified into four groups:

- *Control group* (n = 100): apparently healthy, non-diabetic individuals with a body mass index (BMI) < 25 kg/m^2^.
- *Euglycemic obese group* (n = 100): non-diabetic individuals with BMI ≥ 30 kg/m^2^ and no first-degree family history of diabetes.
- *Obese T2DM group* (n = 100): individuals with T2DM and BMI ≥ 30 kg/m^2^.
- *Non-obese T2DM group* (n = 50): individuals with T2DM and BMI < 25 kg/m^2^.

The diagnosis of T2DM was established according to the American Diabetes Association (ADA) criteria [13]. Exclusion criteria for all participants included type 1 diabetes mellitus, hepatic or renal impairment, familial dyslipidemia, metabolic bone diseases, malignancy, pregnancy, lactation, current smoking, and use of medications known to affect lipid metabolism.

Written informed consent was obtained from all participants prior to enrollment. The study protocol was approved by the Ethics Committee of Misr University for Science and Technology (MUST; FWA00025577) and conducted in accordance with the Declaration of Helsinki.

### Data and sample collection

Following an overnight fast, venous blood samples were collected from all participants. Samples were divided as follows: blood collected in plain tubes was allowed to clot and centrifuged to obtain serum for biochemical analyses, while blood collected in EDTA-containing tubes was used for genomic DNA extraction. A structured questionnaire was administered to collect demographic data (age and sex), anthropometric measurements (weight, height, and BMI), medical history, and information on current medication use.

### Biochemical analysis

Biochemical assessments included fasting blood glucose (FBS), fasting insulin, glycated hemoglobin (HbA1c), and lipid profile parameters (total cholesterol, triglycerides, high-density lipoprotein cholesterol [HDL-C], low-density lipoprotein cholesterol [LDL-C], and triglyceride-to-HDL-C ratio). Insulin resistance was estimated using the homeostasis model assessment of insulin resistance (HOMA-IR) formula [14]:

$${\mathrm{HOMA}} - {\mathrm{IR}} = \frac{{{\text{fasting insulin}} \left( {\mu {\mathrm{U}}/{\mathrm{mL}}} \right) \times {\text{fasting glucose}} \left( {{\mathrm{mmol}}/{\mathrm{L}}} \right)}}{22.5}$$

All biochemical measurements were performed using validated automated analyzers with commercially available kits (Spectrum Diagnostics, Erba Mannheim, and Chemux Bioscience).

### Genotyping analysis

Genomic DNA was extracted from whole blood using the GeneJET Whole Blood Genomic DNA Purification Kit (Thermo Scientific, Waltham, MA, USA). DNA concentration and purity were assessed spectrophotometrically. Genotyping of *APOA1* (rs5069) and *APOE* (rs429358 and rs7412) single-nucleotide polymorphisms was performed using TaqMan^®^ SNP Genotyping Assays (Applied Biosystems, Foster City, CA, USA) according to the manufacturer’s instructions. Real-time PCR amplification was conducted using the QuantStudio™ 5 Real-Time PCR System (Applied Biosystems).

### Statistical analysis

Statistical analyses were performed using IBM SPSS Statistics for Windows (version 26.0) and R software (version 4.5.0; R Foundation for Statistical Computing, Vienna, Austria). The normality of continuous variables was assessed using the Shapiro–Wilk test. Data are presented as mean ± standard deviation (SD) for normally distributed variables and as frequencies and percentages for categorical variables.

Between-group comparisons for continuous variables were conducted using one-way analysis of variance (ANOVA), followed by appropriate post-hoc tests. Genotype and allele frequencies were compared using the chi-square (χ^2^) test. Associations between genotypes and disease outcomes were evaluated by calculating odds ratios (ORs) with 95% confidence intervals (CIs) using binary logistic regression models. Conformity of genotype distributions with Hardy–Weinberg equilibrium (HWE) was assessed using a chi-square test in the control group. Principal component analysis (PCA) was applied to evaluate population stratification. Correlations were assessed using Spearman’s rank correlation coefficient (ρ). All statistical tests were two-tailed, and a *p*-value < 0.05 was considered statistically significant.

## Results

### Demographic and biochemical characteristics

Table 1 summarizes the demographic and biochemical characteristics of the study participants. The cohort (N = 350) comprised four age- and sex-matched groups: healthy controls (n = 100), euglycemic obese individuals (n = 100), obese individuals with T2DM (n = 100), and non-obese individuals with T2DM (n = 50). As defined by the study design, BMI differed significantly across groups (p < 0.001), whereas age and sex distributions were comparable.

**Table 1:** Biochemical data analysis for all cases

Parameters		Control Group (N = 100)	Euglycemic Obese (N = 100)	Obese T2DM (N = 100)	Non-obese T2DM (N = 50)	*p*-value
		Mean (µ ± SD), n%				
Sex, n (%)	Male	29 (29%)	31 (31%)	38 (38%)	22 (44%)	> 0.05
	Female	71 (71%)	69 (69%)	62 (62%)	28 (56%)	
Age (years)		42.7 ± 3.7	43.2 ± 4.6	42.8 ± 5.4	43.3 ± 3.5	> 0.05
BMI (kg/m^2^)		24.6 ± 2.2^a^	35.6 ± 4.7^b^	32.85 ± 3.4^c^	24.7 ± 2.2^a^	< 0.001
FBS (mg/dL)		85.7 ± 9.3^a^	105.6 ± 20.2^b^	131.3 ± 40.6^c^	103.15 ± 23.2^d^	< 0.001
HbA1c (%)		4.7 ± 0.42^a^	5.5 ± 0.7^b^	7.8 ± 1.2^c^	7.4 ± 0.91^d^	< 0.001
Fasting insulin (mU/L)		5.4 ± 3.4^a^	7 ± 4.8^b^	8.2 ± 3.6^c^	7.8 ± 4.3^c, d^	< 0.001
HOMA-IR		1.1 ± 0.7^a^	1.8 ± 1.3^b^	2.6 ± 1.74^c^	1.93 ± 1.01^d^	< 0.001
TC (mg/dL)		152.5 ± 28.2^a^	183.8 ± 38.5^b, c^	181.2 ± 40.1^a, c^	155.9 ± 29.65^d^	< 0.001
TG (mg/dL)		129 ± 33.7^a^	164.1 ± 62.7^b^	148.7 ± 51^c^	106.9 ± 32.07^d^	< 0.001
HDL-C (mg/dL)		55.9 ± 10.2^a^	55.5 ± 8.6^a, b^	50.1 ± 9.1 ^a, c^	55.1 ± 8.4^d^	< 0.001
LDL-C (mg/dL)		70.0 ± 25.3^a^	95.5 ± 33^b^	101.3 ± 37.6 ^a, d^	79.4 ± 30.1^c^	< 0.001
TG/HDL-C ratio		1.96 ± 0.721^a^	3.05 ± 1.3^b^	3.1 ± 1.3^b, c^	2.02 ± 0.83^d^	< 0.001

Values are presented as mean ± standard deviation. Superscript letters (a, b, c) denote significant differences between groups based on post-hoc analysis; groups sharing a common letter are not significantly different.

Analysis of biochemical parameters demonstrated marked metabolic alterations in the patient groups. Serum 25-hydroxyvitamin D [25(OH)D] levels were significantly lower in the euglycemic obese, obese T2DM, and non-obese T2DM groups compared with controls (all p < 0.001). Glycemic indices including fasting blood glucose (FBS), glycated hemoglobin (HbA1c), fasting insulin, and HOMA-IR were significantly elevated in all patient groups relative to controls (all p < 0.001), with the most pronounced derangements observed in obese individuals with T2DM.

Lipid profile analysis revealed significant dyslipidemia in the obese groups (both euglycemic obese and obese T2DM), characterized by higher total cholesterol, triglycerides, low-density lipoprotein cholesterol (LDL-C), and triglyceride-to-HDL-C ratio, alongside lower high-density lipoprotein cholesterol (HDL-C) levels compared with controls (all p < 0.001). In contrast, the non-obese T2DM group exhibited a lipid profile largely comparable to that of the control group.

### Hardy–weinberg equilibrium assessment and population stratification analysis

The genotype distributions of the studied polymorphisms (*APOE* rs429358, rs7412; *APOA1* rs5069) were tested for conformity to Hardy–Weinberg equilibrium (HWE). Deviations from HWE were observed for rs429358 in the control, obese T2DM, and non-obese T2DM groups, and for rs7412 in the obese groups. The *APOA1* rs5069 variant significantly deviated from HWE in all study groups. Supplementary Figure S1 displays these findings.

Principal component analysis (PCA) based on encoded *APOE* and *APOA1* genotypes demonstrated extensive overlap among controls, euglycemic obese, obese T2DM, and non-obese T2DM groups. The first two principal components explained 25% and 18.8% of the variance, respectively, yet showed no distinct clustering by group. The 95% confidence ellipses overlapped substantially, indicating a lack of population substructure within the cohort.

To evaluate the representativeness of our sample, we compared the allele frequencies observed in the control group with available population data from gnomAD and ALFA for individuals of North African and Middle Eastern ancestry. The distribution of *APOE* alleles in our controls, characterized by the predominance of the ε3 allele and lower frequencies of ε2 and ε4, mirrors the pattern reported for regional populations, where ε3 is typically the most common allele (≈70–80%), followed by ε4 (≈10–15%) and ε2 (≈5–10%). Similarly, the allele frequencies of *APOA1* rs5069 in our control group fall within the range documented for Middle Eastern and North African cohorts in public databases. This concordance suggests that our control sample is demographically and genetically comparable to reference populations from the region, supporting the generalizability of our findings.

### Association of APOE and APOA1 rs5069 polymorphisms with disease phenotype

**Fig. 1:**
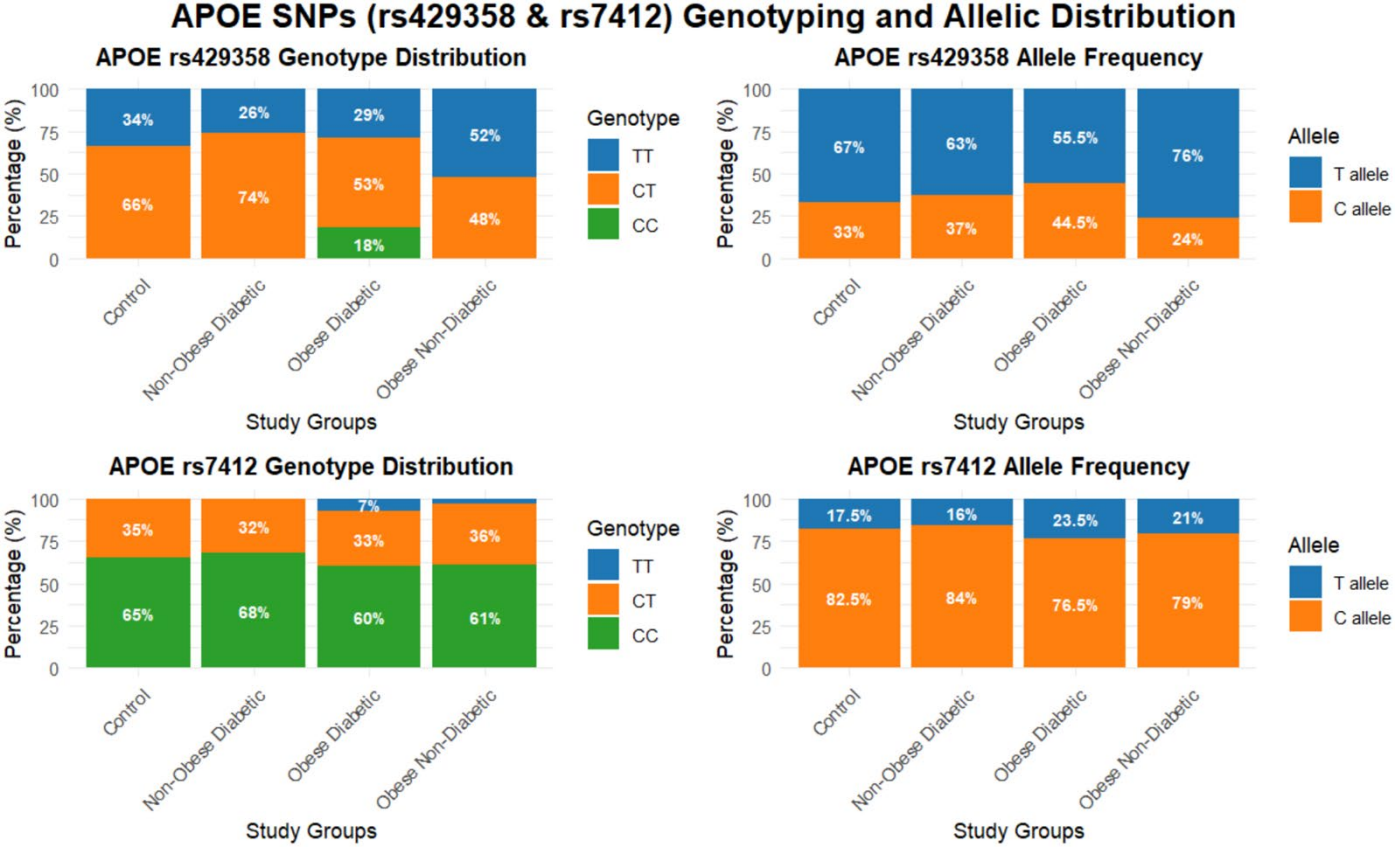
The stacked chart illustrates the frequency distribution of APOE (rs429358–rs7412) genotypes and alleles among the studied groups

Comparative analysis demonstrated significant associations between *APOE* variants and disease status. The rs429358 polymorphism showed robust genotypic differences, particularly between controls and both obese groups (euglycemic and T2DM) (p < 0.001). In contrast, rs7412 showed no significant associations. Figure 1 displays these findings.

**Fig. 2:**
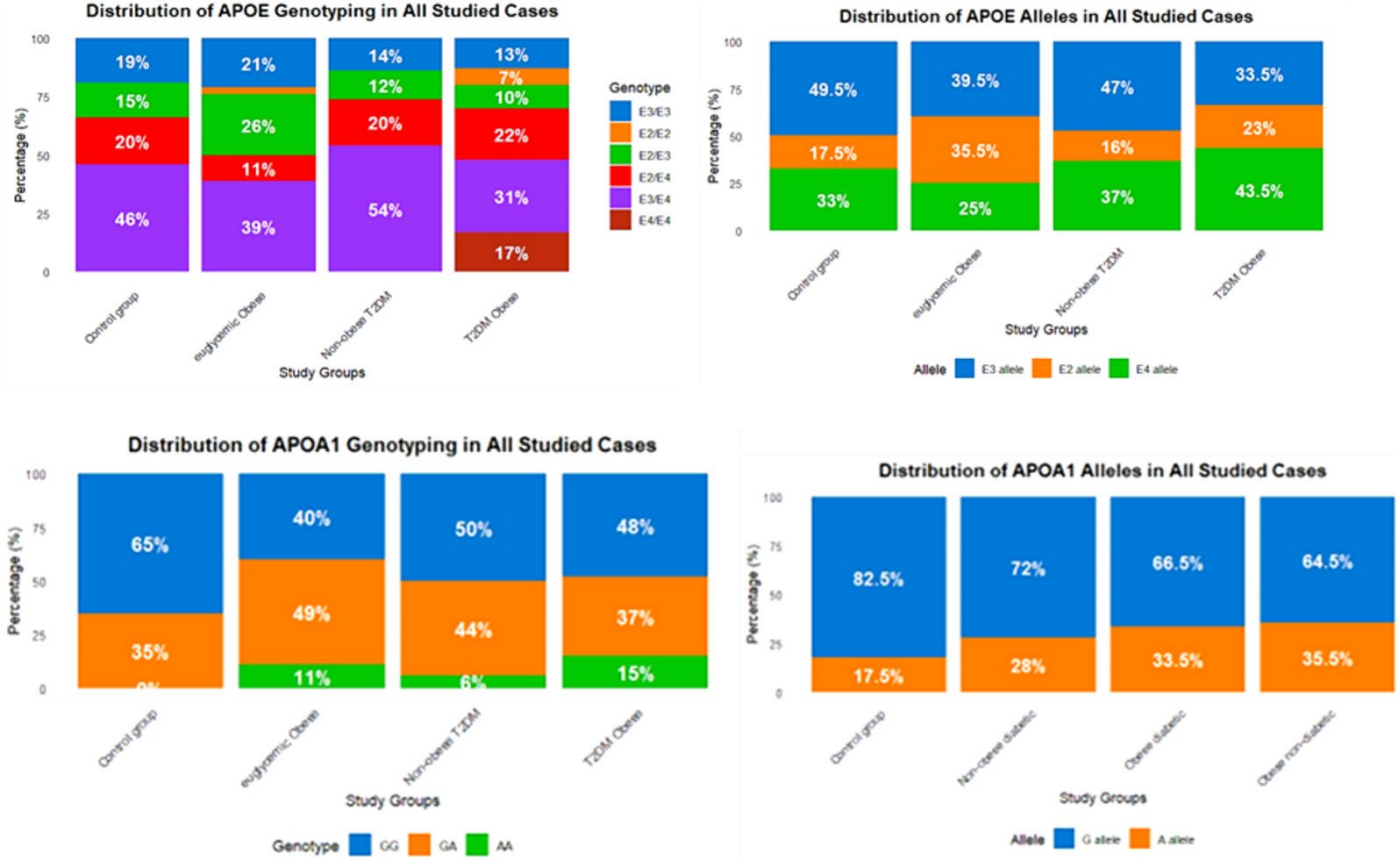
The frequency distribution of *APOE* and *APOA1* rs5069 genotypes and alleles among all studied groups.

Table 2 and Fig. 2 summarize the distribution of *APOE* and *APOA1* (rs5069) genotypes and alleles across the study groups. For *APOE*, significant differences were observed between control and obese groups. The frequency of the E3/E3 genotype declined in obese T2DM subjects (13%) compared with controls (19%) and euglycemic obese individuals (21%) (p < 0.001 and p = 0.005, respectively). The E4/E4 genotype appeared exclusively in the obese T2DM group (17%), contributing to significant group differences. Allele analysis showed higher E2 frequency in euglycemic obese subjects (p < 0.001) and a marked increase in E4 among obese T2DM individuals (p = 0.005 vs. controls; p < 0.001 vs. euglycemic obese).

**Table 2:** The distribution of *APOE* and APOA1 (rs5069) genotypes and alleles in all studied cases

APOE genotyping & alleles	Control group. N = 100	Euglycemic Obese N = 100	Obese T2DM N = 100	Non-obese T2DM N = 50	*P*-value			
					Control versus Euglycemic Obese	Control versus Obese T2DM	Euglycemic Obese versus Obese T2DM	Obese diabetic versus Non-obese T2DM
E3/E3 (ref)	19 (19%)	21 (21%)	13 (13%)	7 (14%)	**0.055**	** < 0.001**	**0.005**	**0.005**
E2/E2	0	3 (3%)	7 (7%)	0 (0%)				
E2/E3	15 (15%)	26 (26%)	10 (10%)	6 (12%)				
E2/E4	20 (20%)	11 (11%)	22 (22%)	10 (20%)				
E3/E4	46 (46%)	39 (39%)	31 (31%)	27 (54%)				
E4/E4	0	0	17 (17%)	0 (0%)				
E3 (ref)	99 (45.5%)	79 (39.5%)	67 (33.5%)	47 (47%)	** < 0.001**	**0.005**	** < 0.001**	0.065
E2	35 (17.5%)	71 (35.5%)	46 (23%)	16 (16%)				
E4	66 (33%)	50 (25%)	87 (43.5%)	37 (37%)				

APOA1 genotyping & alleles. rs5069	Control group. N = 100	Euglycemic Obese N = 100	Obese T2DM N = 100	Non-obese T2DM N = 50	Control versus Euglycemic Obese	Control versus Obese T2DM	Euglycemic Obese versus Obese T2DM	Obese diabetic versus Non-obese T2DM
GG (ref)	65 (65%)	40 (40%)	48 (48%)	25 (50%)	** < 0.001**	**0.011**	** < 0.001**	0.260
GA	35 (35%)	49 (49%)	37 (37%)	22 (44%)				
AA	0	11 (11%)	15 (15%)	3 (6%)				
G (ref)	165 (82.5%)	129 (64.5%)	133 (66.5%)	72 (72%)	** < 0.001**	**0.005**	0.674	0.334
A	35 (17.5%)	71 (35.5%)	67 (33.5%)	28 (28%)				

Bold values indicate significant *p*-values

For *APOA1* rs5069, the GG genotype was more common in controls (65%) than in obese groups, while the AA genotype appeared only in obese subjects (p < 0.001). The A allele frequency increased significantly in both obese groups compared with controls (p < 0.001 and p = 0.005). No significant differences were observed between obese T2DM and non-obese T2DM individuals for both genes. Overall, *APOE* E4-containing genotypes were enriched in obese T2DM subjects, while the *APOA1* rs5069 A allele frequency increased across obese groups irrespective of diabetic status.

**Fig. 3:**
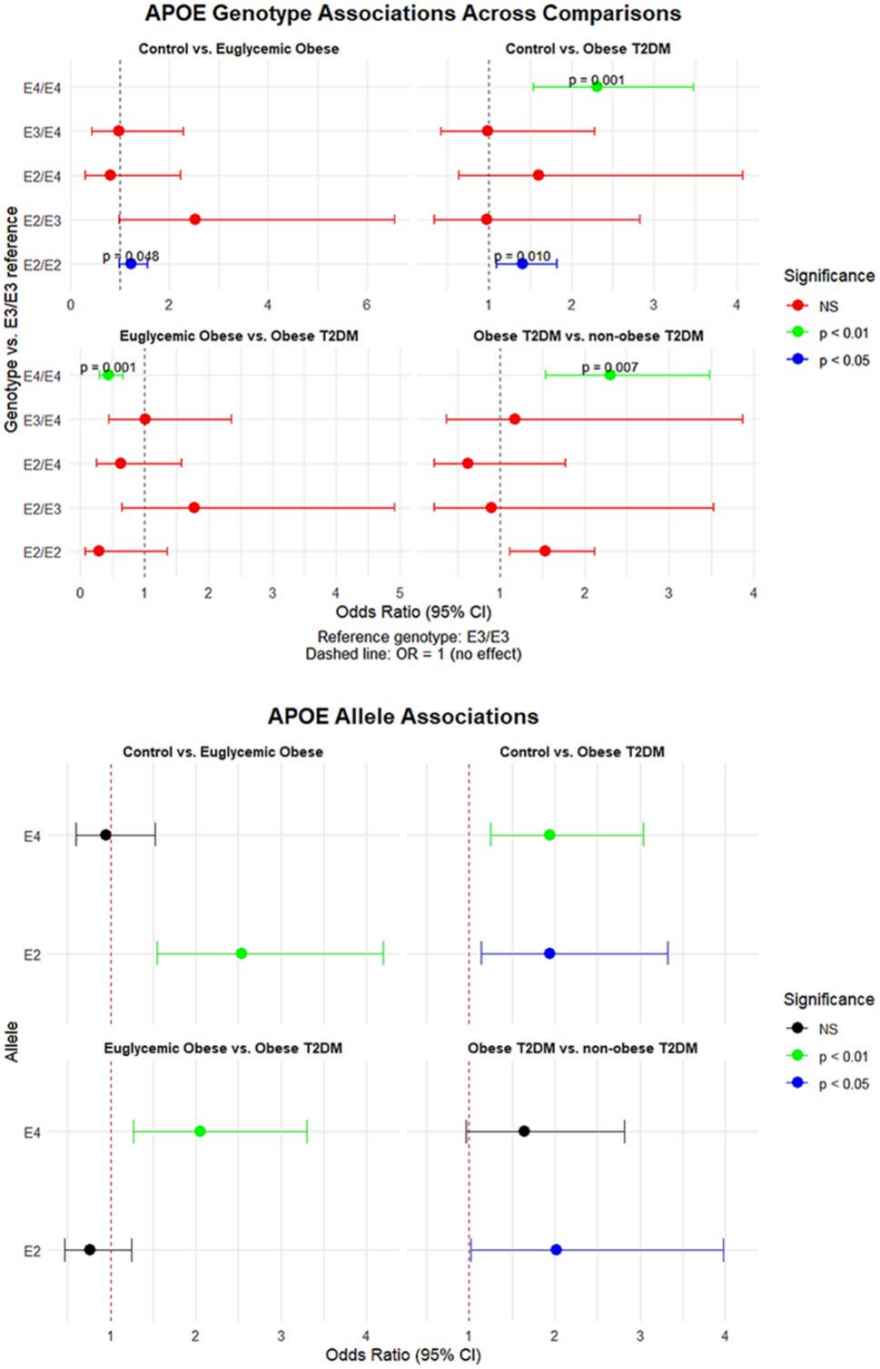
The forest plot illustrates the *APOE* genotypes and alleles association comparison across studied groups

**Fig. 4:**
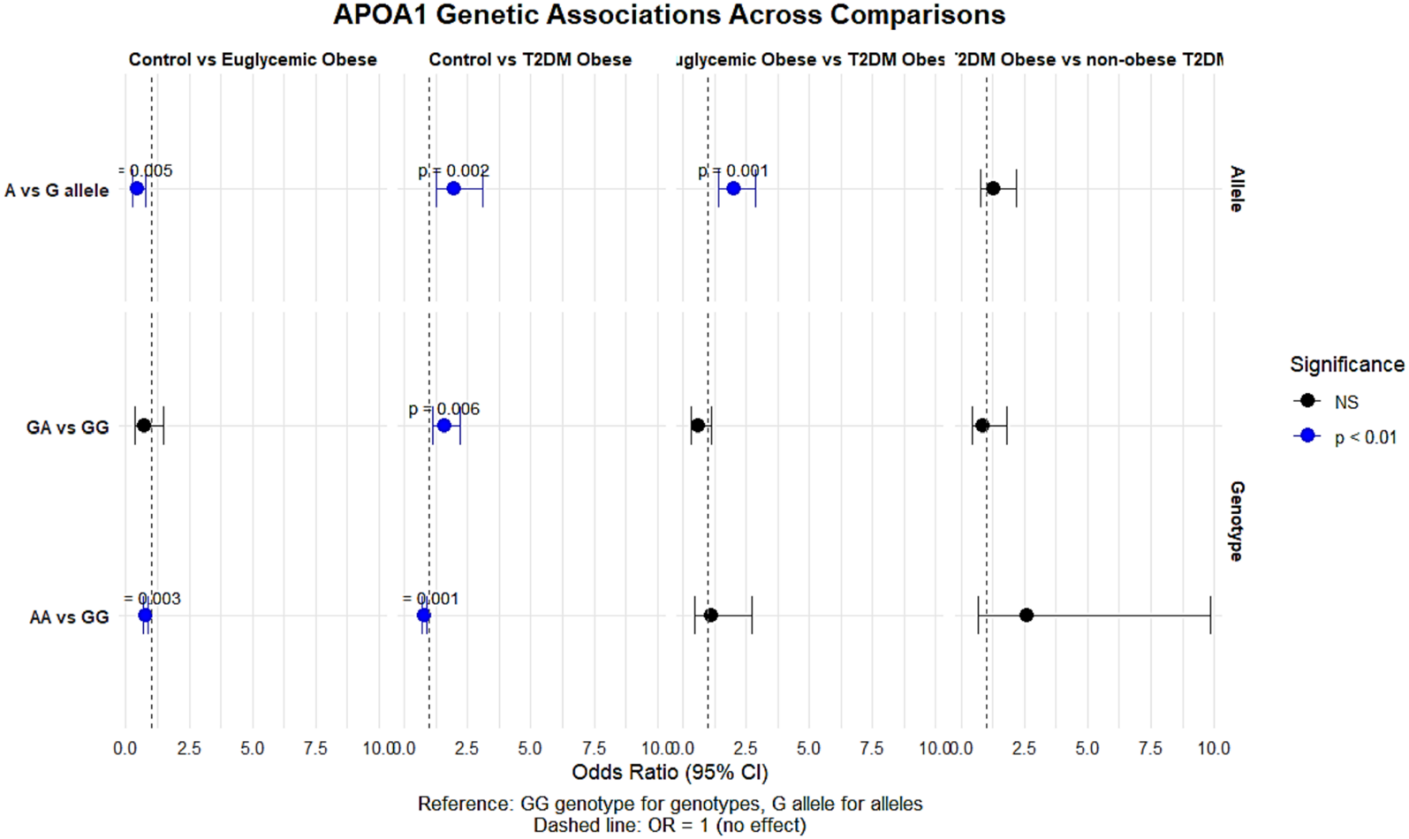
The forest plot illustrates *APOA1* genotypes and alleles association comparison across studied groups

Table 3, Fig. 3 and 4 summarize the univariate associations of *APOE* and *APOA1* genotypes and alleles with obesity and T2DM across several pairwise group comparisons. When comparing controls with euglycemic obese subjects, significant associations were observed for *APOE* variants. The E2/E2 genotype showed a borderline-significant positive association with obesity (OR = 1.231, p = 0.048), and the E2 allele demonstrated a strong association with increased odds of obesity (OR = 2.542, p < 0.001). Conversely, the E4 allele showed no meaningful association in this comparison (p = 0.829). For *APOA1*, the A allele and AA genotype were associated with significantly lower odds of obesity in this comparison (OR = 0.436, p = 0.005 and OR = 0.762, p = 0.003, respectively).

**Table 3:** Associations of *APOE* and *APOA1* (rs5069) genotypes and alleles with the risk of obesity and T2DM compared to controls (univariate analysis)

APOE Genotypes/Alleles	Control versus Euglycemic obese	Control versus obese T2DM	Euglycemic obese versus obese T2DM	Obese T2DM versus non-obese T2DM
	OR (95% CI) *p*-value			
E3/E3 (ref)	–	–	–	–
E2/E2	1.231 (0.973–1.557) **0.048**	1.412 (1.092–1.825) **0.010**	0.293 (0.064–1.348) 0.104	1.538 (1.11–2.122) 0.069
E2/E3	2.533 (0.980–6.547) 0.053	0.974 (0.335–2.831) 0.962	1.779 (0.645–4.907) 0.264	0.897 (0.220–3.520) 0.877
E2/E4	0.804 (0.290–2.228) 0.674	1.608 (0.635–4.074), 0.316	0.622 (0.245–1.576) 0.316	0.618 (0.216–1.773) 0.369
E3/E4	0.985 (0.425–2.281) 0.972	0.985 (0.425–2.281) 0.972	1.015 (0.438–2.351) 0.972	1.185 (0.362–3.873) 0.779
E4/E4	–	2.308 (1.533–3.475) < **0.001**	0.433 (0.288–0.652)** < 0.001**	2.308 (1.533–3.475) **0.007**
E3 (ref)	–	–	–	–
E2	2.542 (1.540–4.197)** < 0.001**	1.942 (1.134–3.326) **0.015**	0.764 (0.466–1.251) 0.284	2.017 (1.022–3.982)** 0.041**
E4	0.949 (0.592–1.522) 0.829	1.948 (1.248–3.041)** 0.003**	2.052 (1.274–3.304)** 0.003**	1.649 (0.965–2.819) 0.066

APOA1 rs5069 Genotype/Allele	Control versus Euglycemic obese	Control versus T2DM obese	Euglycemic obese versus T2DM obese	T2DM obese versus non-obese T2DM
	OR (95% CI) *p*-value			
GG (ref)	–	–	–	–
GA	0.730 (0.355–1.498) 0.390	1.573 (1.135–2.180) 0.006	0.629 (0.346–1.145) 0.128	0.876 (0.428–1.792) 0.717
AA	0.762 (0.664–0.875) **0.003**	0.784 (0.679–0.906) < **0.001**	1.136 (0.469–2.751) 0.777	2.604 (0.688–9.852) 0.148
G (ref)	–	–	–	–
A	0.436 (0.242–0.785) **0.005**	1.972 (1.247–3.118) **0.002**	2.029 (1.424–2.891) < **0.001**	1.295 (0.765–2.192) 0.334

Bold values indicate significant *p*-values

In the comparison between controls and obese subjects with T2DM, a different pattern emerged. The *APOE* E4/E4 genotype demonstrated a significant association with T2DM in obesity (OR = 2.308, p < 0.001), and both the E2 and E4 alleles were associated with higher odds of T2DM-related obesity (E2: OR = 1.942, p = 0.015; E4: OR = 1.948, p = 0.003). For *APOA1*, both the GA genotype (OR = 1.573, p = 0.006) and the A allele (OR = 1.972, p = 0.002) were associated with higher odds of obese T2DM.

When comparing euglycemic obese individuals to obese individuals with T2DM, the E4 allele remained significantly associated with T2DM in the context of obesity (OR = 2.052, p = 0.003), while the E2 allele did not demonstrate a significant association (p = 0.284). For *APOA1*, the A allele showed a significant association with T2DM in obese subjects (OR = 2.029, p < 0.001), although genotype-specific comparisons (GA and AA) did not reach statistical significance.

Finally, comparisons between obese T2DM and non-obese T2DM subjects revealed no statistically significant differences for most *APOE* genotypes or alleles, although the E2 allele showed a modest association (OR = 2.017, p = 0.041). *APOA1* genotypes and alleles did not show significant associations in this comparison, suggesting that rs5069 may be more relevant to obesity-linked metabolic changes than to T2DM independent of adiposity.

### Interplay between APOE and APOA1 polymorphisms

**Fig. 5:**
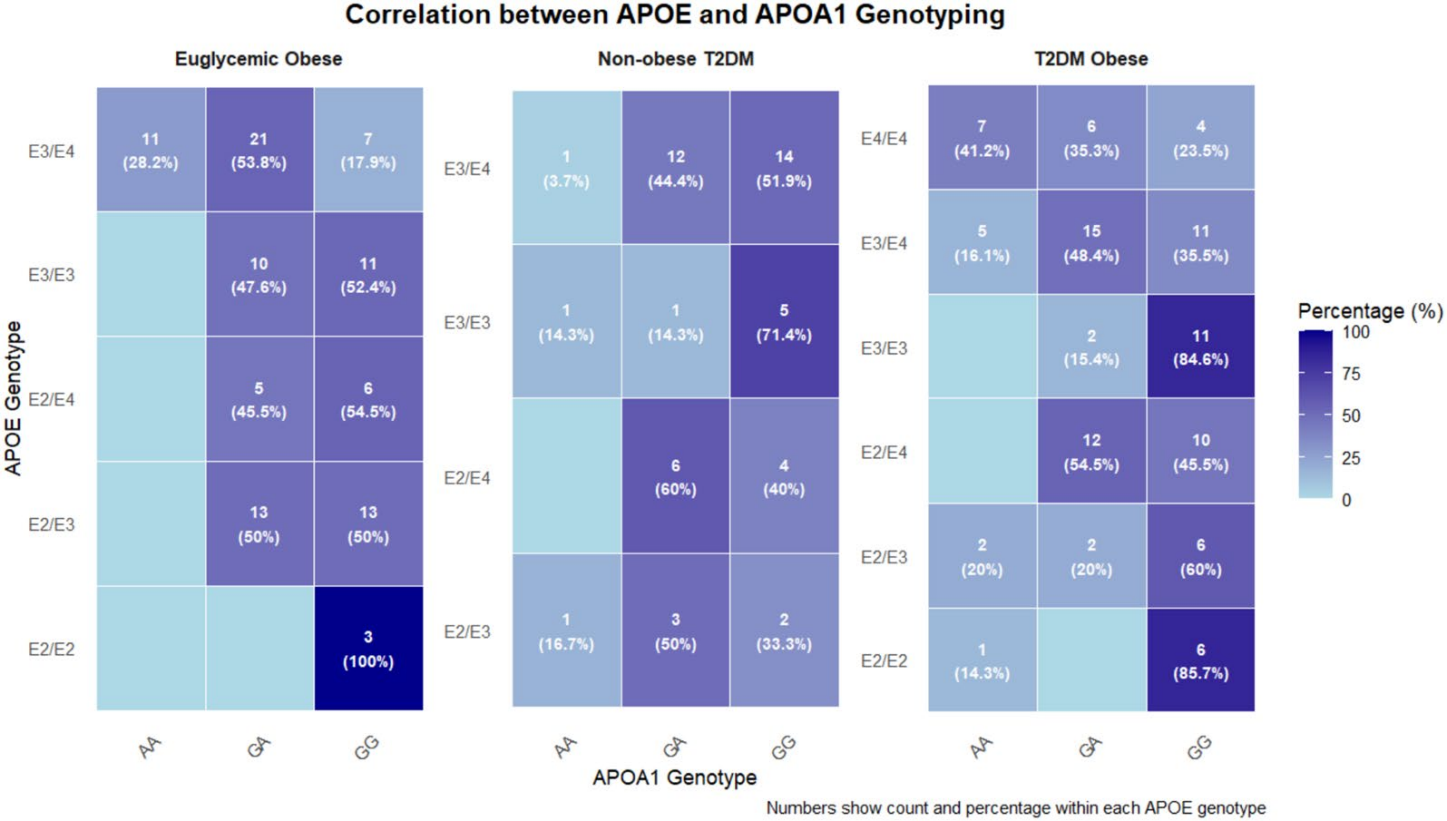
Illustrates the correlation between *APOE* and *APOA1* genotypes in the study groups (euglycemic obese, non-obese T2DM, and obese T2DM). Each tile represents the number of individuals with a given *APOE* genotype (rows) and *APOA1* genotype (columns). The percentages shown in parentheses indicate the proportion of individuals within each *APOE* genotype row who carry the specified *APOA1* genotype (i.e., the percentage of the *APOA1* genotype out of the total count for that *APOE* genotype within the same clinical group). Darker shading indicates a higher percentage. This row-wise percentage representation highlights the distribution of *APOA1* genotypes within each *APOE* background and facilitates comparison of genotype patterns across the three metabolic groups

**Fig. 6:**
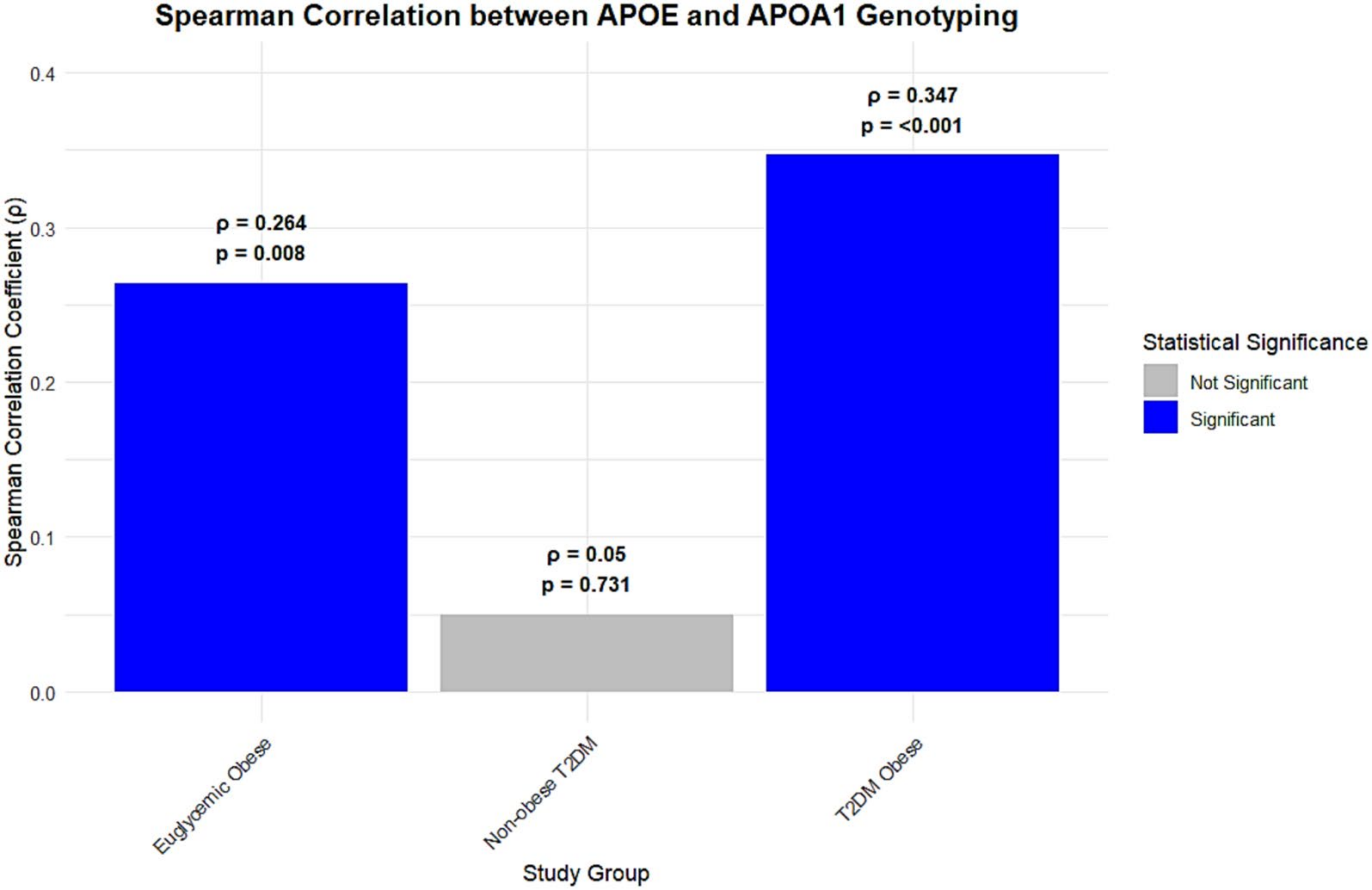
The bar chart illustrates a significant spearman correlation between *APOE* and *APOA1* genotyping across studied groups

The interaction between *APOE* and *APOA1* genotypes was assessed (Table 4, Figs. 5 and 6). A significant positive correlation was observed in both the euglycemic obese (ρ = 0.264, p = 0.008) and obese T2DM (ρ = 0.347, p < 0.001) groups. This correlation was stronger in the presence of T2DM. Conversely, in subgroup analyses, the *APOE* × *APOA1* interaction term was not statistically significant in the non-obese T2DM group. However, given the relatively small sample size of this subgroup (n = 50), the study had limited power to detect interaction effects of modest magnitude. Post hoc power analysis indicated that only large interaction effects (OR ≥ 2.5–3.0) could be detected with adequate power, whereas subtler effects may not have been captured. Therefore, the absence of a detected interaction should be interpreted cautiously and does not exclude the possibility of a biologically meaningful interaction in this subgroup.

**Table 4:** The distribution and correlation between *APOE* genotypes across *APOA1* rs5069 genotypes by study group

Study group	*APOE* genotype	*APOA1* rs5069 genotype			Total	Statistical analysis	
		**GG**	**GA**	**AA**		Chi-Square χ^2^ (*p*-value)	Spearman Correlation ρ (*p*-value)
Euglycemic Obese (N = 100)	E3/E3	11	10	0	21	28.3 **(< 0.001)**	0.264 **(0.008)**
	E2/E2	3	0	0	3		
	E2/E3	13	13	0	26		
	E3/E4	7	21	11	39		
	E2/E4	6	5	0	11		
	Total	40	49	11	100		
T2DM Obese (N = 100)	E3/E3	11	2	0	13	30.2 **(0.001)**	0.347 **(< 0.001)**
	E2/E2	6	0	1	7		
	E2/E3	6	2	2	10		
	E3/E4	11	15	5	31		
	E2/E4	10	12	0	22		
	E4/E4	4	6	7	17		
	Total	48	37	15	100		
Non-Obese T2DM (N = 50)	E3/E3	5	1	1	7	6.01 (0.422)	0.050 (0.731)
	E2/E3	2	3	1	6		
	E3/E4	14	12	1	27		
	E2/E4	4	6	0	10		
	Total	25	22	3	50		

Bold values indicate significant *p*-values

Table 5 shows A multivariate binary logistic regression model was constructed to evaluate the independent and joint effects of *APOE* ε4 carriage, *APOA1* rs5069 A-allele carriage, and their interaction on the risk of T2DM, adjusting for sex, age, and BMI. The model was statistically significant overall (χ^2^ = 13.139, p = 0.041), although it explained a modest proportion of the variance in T2DM risk (Nagelkerke R^2^ = 0.049) and had an overall classification accuracy of 60.3%.

**Table 5:** Multivariate logistic regression model examining the effects of *APOE*, *APOA1*, their interaction, and clinical covariates on T2DM risk

Predictor Variable	B	SE	Wald	*p*-value	OR (Exp B)
*APOA1*_A (A-carrier)	− 0.417	0.402	1.072	0.300	0.659
*APOE*_e4 (ε4-carrier)	0.285	0.315	0.821	0.365	1.330
*APOE* × *APOA1* Interaction	0.726	0.490	2.197	0.138	2.066
Sex	0.456	0.231	3.911	0.048	1.578
Age	− 0.006	0.024	0.058	0.809	0.994
BMI	− 0.001	0.019	0.004	0.952	0.999

*APOE* ε4 carriage (OR = 1.330, p = 0.365) and *APOA1* A-allele status (OR = 0.659, p = 0.300) were not independently associated with T2DM. The *APOE* × *APOA1* interaction term showed a trend toward increased odds of T2DM (OR = 2.066) but did not reach statistical significance (p = 0.138). Among the covariates, sex emerged as a statistically significant predictor (OR = 1.578, p = 0.048), while age and BMI did not contribute significantly to the model.”

## Discussion

This study examined the associations of *APOE* and *APOA1* gene polymorphisms with obesity and type 2 diabetes mellitus (T2DM), with particular attention to their distribution patterns across metabolic phenotypes. Several key observations emerged. First, pronounced dyslipidemia and insulin resistance were confirmed in obese individuals with T2DM, consistent with established metabolic profiles. Second, the *APOE* ε4 allele and ε4/ε4 genotype were significantly associated with T2DM in the context of obesity. Third, the *APOA1* rs5069 A allele and AA genotype were more frequent among obese and diabetic individuals compared with controls. Finally, correlated distribution patterns between *APOE* and *APOA1* genotypes were observed in obese groups but not among non-obese individuals with T2DM, suggesting that adiposity may influence how these genetic variants cluster within metabolic disease states.

All patient groups exhibited significant elevations in glycemic indices (fasting blood glucose, HbA1c, fasting insulin, and HOMA-IR) compared with controls, with obese T2DM participants demonstrating the most severe insulin resistance. Dyslipidemia characterized by elevated total cholesterol, triglycerides, LDL-C, and reduced HDL-C was particularly evident in obese individuals with T2DM. These findings align with previous reports indicating that obesity exacerbates the atherogenic lipid profile associated with T2DM [15, 16]. Clinical and mechanistic studies demonstrate that increased adiposity, especially visceral fat accumulation, amplifies insulin resistance and disrupts lipid metabolism through elevated triglycerides, reduced HDL-C, and increased small dense LDL particles [17]. In contrast, non-obese T2DM participants displayed lipid profiles closer to those of controls, supporting the view that obesity substantially contributes to dyslipidemia in diabetic populations.

Genetic analyses support a role for *APOE* variation in metabolic disease susceptibility. The enrichment of the ε4 allele in obese individuals with T2DM, relative to both controls and euglycemic obese participants, is consistent with findings from multiple populations. Studies in East Asian cohorts, including the Hakka population in southern China, have reported associations between ε4 (rs429358) and increased T2DM risk accompanied by adverse lipid profiles [18]. Similarly, investigations conducted in Egypt [19, 20], Saudi Arabia [21], Lebanon [22], Iran [23], Mexico [24], and Chile [25] have reported higher frequencies of ε4-containing genotypes among individuals with T2DM or dyslipidemia. Meta-analyses further suggest that the ε2 allele may be associated with obesity or altered lipid metabolism in certain populations [26–28], findings supported by studies such as that of Željko et al. [29]. In the present study, the ε2 allele appeared more closely associated with obesity than with overt diabetes, suggesting a potential role in lipid accumulation without marked glycemic dysregulation.

Regarding *APOA1*, the rs5069 A allele and AA genotype were significantly more frequent in obese and diabetic groups compared with controls. Apolipoprotein A-I, which constitutes the majority of HDL protein content, plays a critical role in reverse cholesterol transport and anti-inflammatory processes [30–33]. Variants within the *APOA1* gene have been associated with alterations in HDL levels and functionality across multiple populations. In our cohort, the absence of the AA genotype in controls and its presence in metabolically affected groups suggest that the rs5069 A allele may be linked to less favorable HDL characteristics. Such alterations could be associated with reduced cholesterol efflux capacity and diminished anti-inflammatory effects, features commonly reported in metabolic disease states [34, 36].

The most distinctive observation of this study was the presence of correlated *APOE*–*APOA1* genotype distributions in obese individuals, including both euglycemic obese and obese T2DM groups, but not in non-obese T2DM participants. This pattern suggests that obesity may provide a metabolic context in which coordinated genetic associations related to lipid metabolism become more apparent. One interpretive framework is that the co-occurrence of the *APOE* ε4 allele associated with less efficient clearance of triglyceride-rich lipoproteins and the *APOA1* rs5069 A allele linked to altered HDL properties may coincide with metabolic environments characterized by impaired lipid handling. However, these observations reflect genotypic co-distribution rather than a confirmed synergistic effect on disease risk.

Consistent with this interpretation, multivariate logistic regression analyses adjusting for age, sex, and BMI did not demonstrate a statistically significant *APOE* × *APOA1* interaction on T2DM risk. Although the interaction term yielded an odds ratio greater than two, this estimate did not reach statistical significance (p = 0.138), likely reflecting limited statistical power to detect interaction effects in a moderate-sized cohort. The modest explained variance (Nagelkerke R^2^ = 0.049) indicates that clinical and metabolic factors remain the principal determinants of overt T2DM status, whereas genetic variation may be more relevant earlier in the metabolic trajectory, particularly in shaping lipid metabolism, adiposity, and insulin resistance. The observed association with sex further underscores the contribution of biological and hormonal factors to diabetes susceptibility.

From a mechanistic perspective, obesity is increasingly recognized as a state of chronic low-grade inflammation marked by adipocyte hypertrophy, macrophage infiltration, dysregulated adipokine secretion, and elevated circulating free fatty acids [35–37]. *APOE* is highly expressed in adipose tissue and participates in triglyceride-rich lipoprotein clearance and immunometabolism regulation. Experimental models have shown that *APOE* influences adipose tissue expansion, macrophage activation, and inflammasome signaling [38]. The ε4 isoform has been associated with a more atherogenic lipoprotein profile and heightened inflammatory responses [39], features that may be particularly relevant in obesogenic environments. Similarly, *APOA1*and functional HDL exert anti-inflammatory and antioxidant effects through cholesterol efflux and modulation of macrophage activity [40]. Variants such as *APOA1* rs5069 have been linked to altered *APOA1* expression and HDL function [41], which in obese individuals may coincide with reduced capacity to counterbalance lipid accumulation and inflammation.

Several limitations of our study should be considered when interpreting the findings. First, deviations from Hardy–Weinberg equilibrium (HWE) were observed for *APOE* rs429358 in multiple groups and for *APOA1* rs5069 across all study categories. To ensure data reliability, we conducted genotyping quality control, including re-genotyping approximately 5% of randomly selected samples, which demonstrated 100% concordance. Principal component analysis (PCA) of the genotype data also showed no evidence of population stratification within the cohort. Importantly, the deviation from HWE for *APOA1* rs5069 observed in the control group represents a methodological limitation, as departures from HWE in healthy controls are suboptimal even in the absence of detectable stratification or technical error. While HWE deviations in disease-enriched subgroups may arise from non-random sampling and true genotype–phenotype associations, this issue underscores the need for cautious interpretation of the *APOA1* findings and for independent replication in larger, population-based cohorts. Second, the relatively small sample size of the non-obese T2DM group may have reduced statistical power for detecting associations in this subgroup. Third, the case control design precludes establishing causal relationships and restricts interpretation to associations.

Future studies should aim to replicate these findings in larger, ethnically diverse populations and, ideally, through longitudinal designs to clarify temporal relationships between these polymorphisms, obesity, and T2DM. Functional and multi-omics investigations are also needed to elucidate the biological pathways underlying the context-dependent genetic effects observed here and to examine gene–environment interactions that may refine metabolic risk stratification.

**In conclusion**, this study identifies significant associations between the *APOE* ε4 allele and T2DM in obese individuals, as well as between the *APOA1* rs5069 A allele and both obesity and T2DM. Although *APOE* and *APOA1* genotypes showed correlated distribution patterns in obese groups, the formal gene–gene interaction did not reach statistical significance in multivariable analyses. These findings suggest that obesity may act as a contextual modifier of genetic associations related to lipid metabolism and insulin resistance. Larger, well-powered, and longitudinal studies are needed to determine whether true biological interaction exists between these loci.

## Supplementary Information

Additional file 1

## Data Availability

All data generated or analyzed during this study are included in this article.

## References

[1] Chandrasekaran P, Weiskirchen R. The role of obesity in type 2 diabetes mellitus—an overview. Int J Mol Sci. 2024;25(3):1882. 38339160 10.3390/ijms25031882 PMC10855901

[2] Lu X, Xie Q, Pan X, Zhang R, Zhang X, Peng G, et al. Type 2 diabetes mellitus in adults: pathogenesis, prevention and therapy. Signal Transduct Target Ther. 2024;9(1):262. 39353925 10.1038/s41392-024-01951-9 PMC11445387

[3] Albitar O, D’Souza CM, Adeghate EA. Effects of lipoproteins on metabolic health. Nutrients. 2024;16(13):2156. 38999903 10.3390/nu16132156 PMC11243180

[4] Matsunaga A, Saito T. Impact of Apolipoprotein E variants: a review of naturally occurring variants and clinical features. J Atheroscler Thromb. 2025;32(3):281–303. 39779225 10.5551/jat.65393 PMC11883201

[5] Seripa D, D’Onofrio G, Panza F, Cascavilla L, Masullo C, Pilotto A. The genetics of the human APOE polymorphism. Rejuvenation Res. 2011;14(5):491–500. 10.1089/rej.2011.1169. 21958003 10.1089/rej.2011.1169

[6] Bhale AS, Venkataraman K. Leveraging knowledge of HDLs major protein ApoA1: structure, function, mutations, and potential therapeutics. Biomed Pharmacother. 2022;154:113634. 36063649 10.1016/j.biopha.2022.113634

[7] Casillas FA, Fernández DEM, Valle Y, Ramírez MA, Parra-Reyna B, Pulido SS, et al. APOA1 (-75 G> A and 83 C> T) and APOB (2488 C> T) polymorphisms and their association with myocardial infarction, lipids and apolipoproteins in patients with type 2 diabetes mellitus. Arch Med Sci AMS. 2021;18(6):1438. 36457989 10.5114/aoms/108674 PMC9710253

[8] Islam MS, Wei P, Suzauddula M, Nime I, Feroz F, Acharjee M, et al. The interplay of factors in metabolic syndrome: understanding its roots and complexity. Mol Med. 2024;30(1):279. 39731011 10.1186/s10020-024-01019-y PMC11673706

[9] Marrades MP, Gonzalez-Muniesa P, Martínez JA, Moreno-Aliaga MJ. A dysregulation in CES1, APOE and other lipid metabolism-related genes is associated to cardiovascular risk factors linked to obesity. Obes Facts. 2010;3(5):312–8. 20975297 10.1159/000321451 PMC6452131

[10] Chaudhary R, Likidlilid A, Peerapatdit T, Tresukosol D, Srisuma S, Ratanamaneechat S, et al. Apolipoprotein E gene polymorphism: effects on plasma lipids and risk of type 2 diabetes and coronary artery disease. Cardiovasc Diabetol. 2012;11(1):36. 22520940 10.1186/1475-2840-11-36 PMC3372424

[11] Krishnamurthy HK, Rajavelu I, Reddy S, Pereira M, Jayaraman V, Krishna K, et al. Association of apolipoprotein E (APOE) polymorphisms with serological lipid and inflammatory markers. Cureus. 2024;16(5):e60721. 10.7759/cureus.60721. (**PMID: 38903305; PMCID: PMC11187349**). 38903305 10.7759/cureus.60721 PMC11187349

[12] Osei-Hwedieh DO, Amar M, Sviridov D, Remaley AT. Apolipoprotein mimetic peptides: mechanisms of action as anti-atherogenic agents. Pharmacol Ther. 2011;130(1):83–91. 10.1016/j.pharmthera.2010.12.003. (**Epub 2010 Dec 21. PMID: 21172387; PMCID: PMC3043134**). 21172387 10.1016/j.pharmthera.2010.12.003 PMC3043134

[13] American Diabetes Association. Blood glucose & A1C: Understanding diabetes diagnosis. Accessed: Jun. 2024;28.

[14] Placzkowska S, Pawlik-Sobecka L, Kokot I, Piwowar A. Indirect insulin resistance detection: current clinical trends and laboratory limitations. Biomed Pap. 2019;163(3):187–99. 10.5507/bp.2019.021 31165793

[15] Bays HE, Kirkpatrick CF, Maki KC, Toth PP, Morgan RT, Tondt J, et al. Obesity, dyslipidemia, and cardiovascular disease: a joint expert review from the Obesity Medicine Association and the National Lipid Association 2024. J Clin Lipidol. 2024;18(3):e320–50. 38664184 10.1016/j.jacl.2024.04.001

[16] Feingold KR. (Updated 2023 Jun 19). Obesity and Dyslipidemia. In: Feingold KR, Ahmed SF, Anawalt B, et al., editors. Endotext [Internet]. South Dartmouth (MA): MDText.com, Inc.; 2000-. Available from: https://www.ncbi.nlm.nih.gov/books/NBK305895//?utm_source=chatgpt.com.

[17] Taresh AI. Assessment of lipid profile and clinical manifestation of obese patients with type 2 diabetes. Eur J Med Health Res. 2024;2(4):105–10.

[18] Deng X, Hou J, Deng Q, Zhong Z. Association between the APOE gene polymorphism and lipid profile and the risk of atrial fibrillation. Lipids Health Dis. 2021;20(1):123. 34587962 10.1186/s12944-021-01551-4 PMC8482687

[19] Galal AA, Abd Elmajeed AA, Elbaz RA, Wafa AM, Elshazli RM. Association of apolipoprotein E gene polymorphism with the risk of T2DM and obesity among Egyptian subjects. Gene. 2021;769:145223. 33059023 10.1016/j.gene.2020.145223

[20] Atta MI, Abo Gabal K, El-Hadidi K, Swellam M, Genina A, Zaher NF. Apolipoprotein E genotyping in Egyptian diabetic nephropathy patients. IUBMB Life. 2016;68(1):58–64. 26662731 10.1002/iub.1460

[21] Alharbi KK, Syed R, Alharbi FK, Khan IA. Association of apolipoprotein E polymorphism with impact on overweight University pupils. Genet Test Mol Biomarkers. 2017;21(1):53–7. 10.1089/gtmb.2016.0190. (**PMID: 28085496**). 28085496 10.1089/gtmb.2016.0190

[22] Atageldiyeva KK, Nemr R, Echtay A, Racoubian E, Sarray S, Almawi WY. Apolipoprotein E genetic polymorphism influence the susceptibility to nephropathy in type 2 diabetes patients. Gene. 2019;715:144011. 10.1016/j.gene.2019.144011. (**Epub 2019 Jul 26. PMID: 31357022**). 31357022 10.1016/j.gene.2019.144011

[23] Karimoei M, Pasalar P, Mehrabzadeh M, Daneshpour M, Shojaee M, Forouzanfar K, et al. Association between apolipoprotein E polymorphism and nephropathy in Iranian diabetic patients. Saudi J Kidney Dis Transpl. 2017;28(5):997–1002. 10.4103/1319-2442.215137. (**PMID: 28937055**). 28937055 10.4103/1319-2442.215137

[24] Santos A, Salguero ML, Gurrola C, Muñoz F, Roig-Melo E, Panduro A. The e4 allele of apolipoprotein E gene is a potential risk factor for the severity of macular edema in type 2 diabetic Mexican patients. Ophthalmic Genet. 2002;23(1):13–9. 10.1076/opge.23.1.13.2203. (**PMID: 11910554**). 11910554 10.1076/opge.23.1.13.2203

[25] Leiva E, Mujica V, Elematore I, Orrego R, Díaz G, Prieto M, et al. Relationship between apolipoprotein E polymorphism and nephropathy in type-2 diabetic patients. Diabetes Res Clin Pract. 2007;78(2):196–201. 10.1016/j.diabres.2007.03.018. (**Epub 2007 May 3. PMID: 17481771**). 17481771 10.1016/j.diabres.2007.03.018

[26] Yin YW, Qiao L, Sun QQ, Hu AM, Liu HL, Wang Q, et al. Influence of apolipoprotein E gene polymorphism on development of type 2 diabetes mellitus in Chinese Han population: a meta-analysis of 29 studies. Metabolism. 2014;63(4):532–41. 10.1016/j.metabol.2013.12.008. (**Epub 2013 Dec 18. PMID: 24439487**). 24439487 10.1016/j.metabol.2013.12.008

[27] Chen DW, Shi JK, Li Y, Yang Y, Ren SP. Association between ApoE polymorphism and type 2 diabetes: a meta-analysis of 59 studies. Biomed Environ Sci. 2019;32(11):823–38. 10.3967/bes2019.104. (**PMID: 31910940**). 31910940 10.3967/bes2019.104

[28] Anthopoulos PG, Hamodrakas SJ, Bagos PG. Apolipoprotein E polymorphisms and type 2 diabetes: a meta-analysis of 30 studies including 5423 cases and 8197 controls. Mol Genet Metab. 2010;100(3):283–91. 10.1016/j.ymgme.2010.03.008. (**Epub 2010 Mar 19. PMID: 20381392**). 20381392 10.1016/j.ymgme.2010.03.008

[29] Zeljko HM, Škarić-Jurić T, Narančić NS, Tomas Ž, Barešić A, Salihović MP, et al. E2 allele of the apolipoprotein E gene polymorphism is predictive for obesity status in Roma minority population of Croatia. Lipids Health Dis. 2011;10(1):9. 10.1186/1476-511X-10-9. (**PMID: 21244662; PMCID: PMC3025844**). 21244662 10.1186/1476-511X-10-9 PMC3025844

[30] Blangero J, Williams-Blangero S, Mahaney MC, Comuzzie AG, Hixson JE, Samollow PB, et al. Effects of a major gene for apolipoprotein A-I concentration are thyroid hormone dependent in Mexican Americans. Arterioscler Thromb Vasc Biol. 1996;16(9):1177–83. 10.1161/01.atv.16.9.1177. (**PMID: 8792772**). 8792772 10.1161/01.atv.16.9.1177

[31] Liao B, Cheng K, Dong S, Liu H, Xu Z. Effect of apolipoprotein A1 genetic polymorphisms on lipid profiles and the risk of coronary artery disease. Diagn Pathol. 2015;10(1):102. 10.1186/s13000-015-0328-7. (**PMID: 26173491; PMCID: PMC4502599**). 26173491 10.1186/s13000-015-0328-7 PMC4502599

[32] Serrano NC, Rojas LZ, Gamboa-Delgado EM, Suárez DP, Salazar Acosta I, Romero SL, et al. Efficacy of vitamin D supplementation in reducing body mass index and lipid profile in healthy young adults in Colombia: a pilot randomised controlled clinical trial. J Nutr Sci. 2023;12:e29. 10.1017/jns.2022.108. (**PMID: 36843975; PMCID: PMC9947753**). 36843975 10.1017/jns.2022.108 PMC9947753

[33] Kim MR, Jeong SJ. Relationship between vitamin D level and lipid profile in non-obese children. Metabolites. 2019;9(7):125. 10.3390/metabo9070125. (**PMID: 31262034; PMCID: PMC6680594**). 31262034 10.3390/metabo9070125 PMC6680594

[34] Butnariu LI, Gorduza EV, Țarcă E, Pânzaru MC, Popa S, Stoleriu S, et al. Current data and new insights into the genetic factors of atherogenic dyslipidemia associated with metabolic syndrome. Diagnostics. 2023;13(14):2348. 10.3390/diagnostics13142348. (**PMID: 37510094; PMCID: PMC10378477**). 37510094 10.3390/diagnostics13142348 PMC10378477

[35] Jung UJ, Choi MS. Obesity and its metabolic complications: the role of adipokines and the relationship between obesity, inflammation, insulin resistance, dyslipidemia and nonalcoholic fatty liver disease. Int J Mol Sci. 2014;15(4):6184–223. 10.3390/ijms15046184. (**PMID: 24733068; PMCID: PMC4013623**). 24733068 10.3390/ijms15046184 PMC4013623

[36] Stryjecki C, Mutch DM. Fatty acid–gene interactions, adipokines and obesity. Eur J Clin Nutr. 2011;65(3):285–97. 21224869 10.1038/ejcn.2010.277

[37] Kojta I, Chacińska M, Błachnio-Zabielska A. Obesity, bioactive lipids, and adipose tissue inflammation in insulin resistance. Nutrients. 2020;12(5):1305. 10.3390/nu12051305. (**PMID: 32375231; PMCID: PMC7284998**). 32375231 10.3390/nu12051305 PMC7284998

[38] Zhang Y, Cheng Z, Hong L, Liu J, Ma X, Wang W, et al. Apolipoprotein E (ApoE) orchestrates adipose tissue inflammation and metabolic disorders through NLRP3 inflammasome. Mol Biomed. 2023;4(1):47. 10.1186/s43556-023-00158-8. (**PMID: 38062308; PMCID: PMC10703753**). 38062308 10.1186/s43556-023-00158-8 PMC10703753

[39] Cash JG, Kuhel DG, Basford JE, Jaeschke A, Chatterjee TK, Weintraub NL, et al. Apolipoprotein E4 impairs macrophage efferocytosis and potentiates apoptosis by accelerating endoplasmic reticulum stress. J Biol Chem. 2012;287(33):27876–84. 10.1074/jbc.M112.377549. (**Epub 2012 Jun 23. PMID: 22730380; PMCID: PMC3431692**). 22730380 10.1074/jbc.M112.377549 PMC3431692

[40] Domínguez-Díaz C, Morán-Moguel MC, Navarro-Hernandez RE, Romo-Vázquez R, Mendizabal-Ruiz AP. Association of SNP rs5069 in APOA1 with benign breast diseases in a Mexican population. Genes. 2022;13(5):738. 10.3390/genes13050738. 35627123 10.3390/genes13050738 PMC9141650

[41] Feng DW, Ma RL, Guo H, He J, Yan YZ, Muratbek, Niu Q, Li SG, Rui DS, Sun F, Zhang M, Zhang JY, Ding YS, Liu JM, Wang K, Guo SX. Association of APOA1 gene polymorphisms (rs670, rs5069, and rs2070665) with dyslipidemia in the Kazakhs of Xinjiang. Genet Mol Res. 2016. 10.4238/gmr.15028094. PMID: 27173266. 10.4238/gmr.15028094 27173266

