# Supplementary material for: The interplay of APOE and APOA1 gene polymorphisms modulates the risk of type 2 diabetes mellitus in an obese population: a case–control study"
Source: Eur J Med Res. 2026 Jan 27;31:155. doi: 10.1186/s40001-025-03829-0 (PMC12849122; doi:10.1186/s40001-025-03829-0)
Supplement: Supplementary file 1 — Additional file 1 [file 40001_2025_3829_MOESM1_ESM.docx]

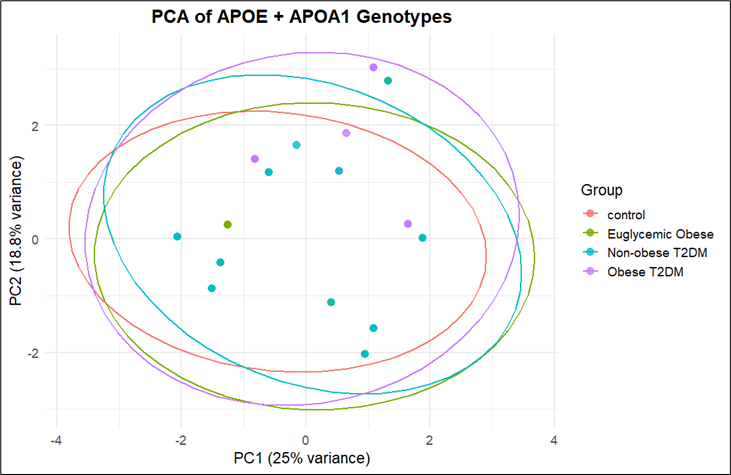


Supplementary Figure 1. PCA of APOE and APOA1 genotypes. The plot depicts the first two principal components (PC1 = 25%, PC2 = 18.8% of total genetic variance). Colors represent the four study groups, and ellipses indicate the 95% confidence intervals for each group. No separation or clustering was observed, indicating no evidence of population stratification.
